# Supplementary material for: Prospective Assessment of Tumour Burden and Bone Disease in Plasma Cell Dyscrasias Using DW-MRI and Exploratory Bone Biomarkers
Source: Cancers (Basel). 2022 Dec 23;15(1):95. doi: 10.3390/cancers15010095 (PMC9817825; doi:10.3390/cancers15010095)
Supplement: Supplementary file 1 [file cancers-15-00095-s001.zip › cancers-2032661-supplementary.pdf]

cancers

MDPI

Supplementary Materials: Prospective Assessment of Tumour Burden and Bone Disease in Plasma Cell Dyscrasias Using DW-MRI and Exploratory Bone Biomarkers

Gaurav Agarwal, Guido Nador, Sherin Varghese, Hiwot Getu, Charlotte Palmer, Edmund Watson, Claudio Pereira, Germana Sallemi, Karen Partington, Neel Patel, Rajkumar Soundarajan, Rebecca Mills, Richard Brouwer, Marina Maritati, Aarti Shah, Delia Peppercorn, Udo Oppermann, Claire M. Edwards, Christopher T. Rodgers, Muhammad Kassim Javaid, Sarah Gooding and Karthik Ramasamy

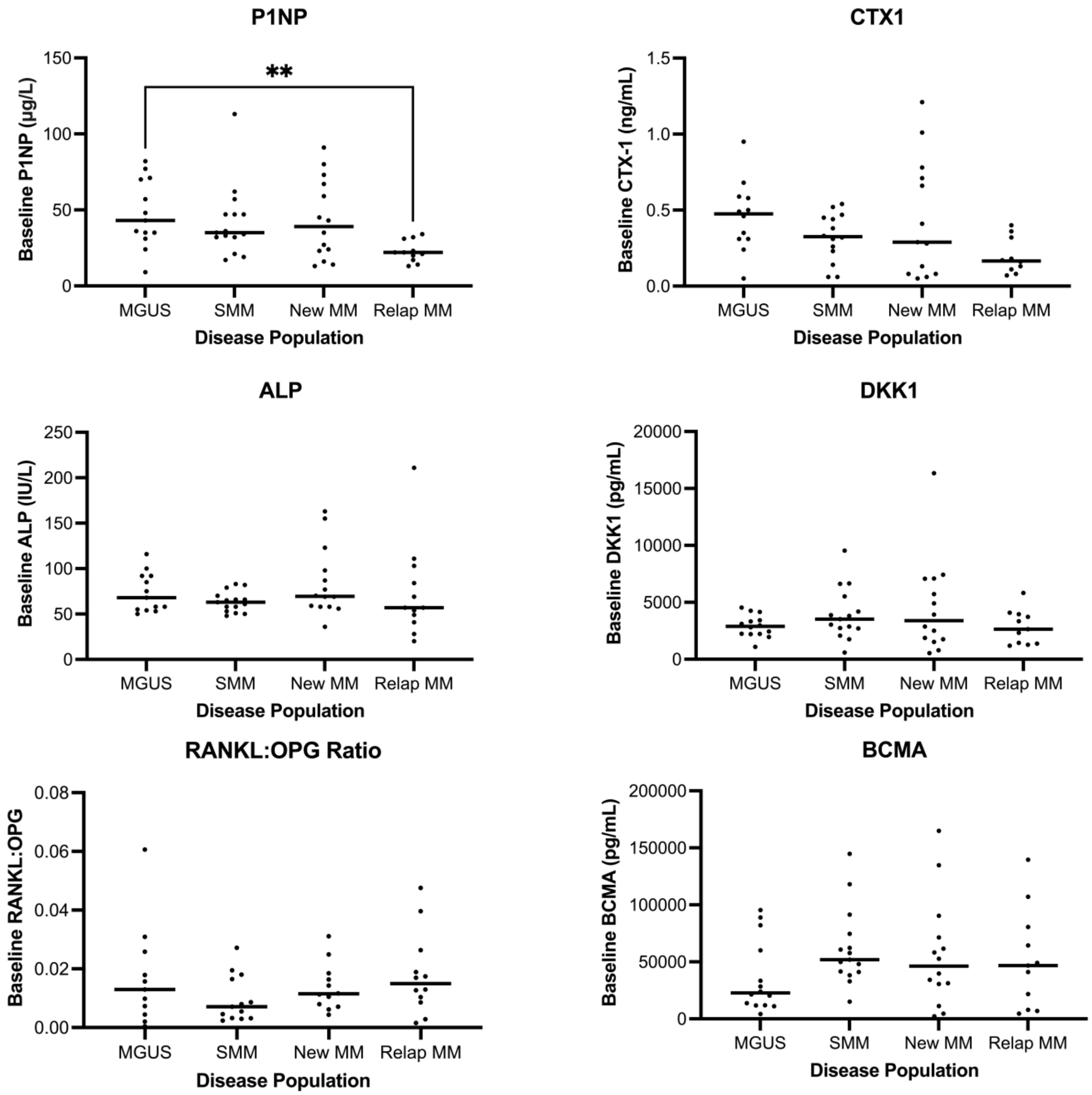

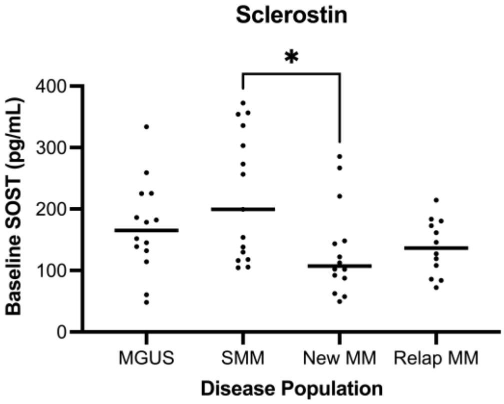

Figure S1. Baseline serum biomarker measurements by disease population.
